# Cloning and rescue of the genome of *Bombyx mori* bidensovirus, and characterization of a recombinant virus

**DOI:** 10.1186/s12985-016-0576-5

**Published:** 2016-07-08

**Authors:** Panpan Zhang, Di Miao, Yahui Zhang, Meizi Wang, Zhaoyang Hu, Peng Lü, Qin Yao

**Affiliations:** Institute of Life Sciences, Jiangsu University, 301 Xuefu Road, Zhenjiang, 212013 China

**Keywords:** *Bombyx mori* bidensovirus, genome clone, linear co-transfection, virus rescue

## Abstract

**Background:**

*Bombyx mori* bidensovirus (BmBDV), which belongs to the *Bidnaviridae* family established by the International Committee on Taxonomy of Viruses in 2011, was the first bidensovirus identified in insects. The structure of BmBDV is similar to that of parvoviruses, while its replication is similar to that of adenoviruses. Although BmBDV has the potential to be used as a tool in biological pest control and as an expression vector, virus rescue has been a bottleneck in the application of this virus.

**Methods:**

In this study, we constructed a full-length genomic clone of BmBDV and showed that its terminal structure was restored. A recombinant BmBDV that expressed the green fluorescence protein (GFP) gene was constructed. Then, *Bm*N cells, which are an ovarian cell line, were co-transfected with the linearized genome using continuous culture and expanded cell culture methods.

**Results:**

The results showed that the GFP gene was expressed successfully, and that cell lesions occurred in virus-infected cells. Furthermore, typical densonucleosis viruses were observed in reinfected silkworm larvae and larval midgut tissues infected by BmBDV, as evidenced by the emission of green fluorescence.

**Conclusions:**

Overall, these results suggest that the virus could be rescued from the infected *Bm*N cells after co-transfection with the linear full length virus genome.

## Background

Bombyx mori bidensovirus (BmBDV) can specifically infect the columnar cells of the midgut epithelium [1] of silkworms, and it causes chronic densonucleosis disease, which results in serious economic losses to the sericulture industry. BmBDV was previously named *B. mori* densovirus type 2 (BmDNV-2) [2] and belonged to the subfamily *Densovirinae* of the family *Parvoviridae*. Just like *B. mori* densovirus type 1 (BmDNV-1) and other parvoviruses, BmBDV is a non-enveloped virus that has a spherical, icosahedral structure that is approximately 22 nm in diameter. However, the BmBDV genome contains two non-homologous, single-stranded linear DNA molecules (VD1 and VD2; 6,543 and 6,024 nucleotides (nts), respectively), which are encapsidated into separate virions [3]. Both VD1 and VD2 DNAs have inverted terminal repeats (ITRs) and share a common terminal sequence (CTS) of 53 nts [2]. However, unlike other parvoviruses, VD1 and VD2 do not contain terminal palindromic sequences that can form terminal hairpins [4]. Instead, the ITRs form a “panhandle structure” [5, 6]. A Basic Local Alignment Search Tool analysis revealed that VD1 contains a highly conserved domain of B family DNA polymerase [7, 8]. In addition, BmBDV is the only virus that carries a single-stranded DNA that encodes a DNA polymerase. In consideration of the specificity of this virus, in 2011, the International Committee on the Taxonomy of Viruses established a new family, *Bidnaviridae*, and designated BmBDV as the type species in the new genus *Bidensovirus* [9] (Table 1).

There are two strains of BmBDV, the Japanese Yamanashi strain and the Chinese Zhenjiang strain. The whole genome sequence of BmBDV (a Zhenjiang isolate) was completely sequenced and submitted to GenBank in 2005. VD1 was 6,543 nts long and VD2 was 6,022 nts long (accession nos. DQ017268 and DQ017269, respectively) [10, 11]. Analyses of the BmBDV genome revealed that there are four open reading frames (ORFs) in VD1, which encode nonstructural protein 2 (NS2, VD1-ORF1) [12], nonstructural protein 1 (NS1, VD1-ORF2) [11], the major structural protein VP (VD1-ORF3) [13], and DNA polymerase (VD1-ORF4) [7], respectively. In contrast, VD2 contains two ORFs that encode nonstructural protein 3 (NS3, VD2-ORF1) [5] and the minor capsid structural protein (mCP) (VD2-ORF2) [13].

The BmBDV virus is similar to parvoviruses, but it has a bipartite genome that replicates differently than other parvoviruses. The termini of the BmBDV genome lack palindromic sequences [4]. To elucidate the difference in the replication mechanism between *Bm*DNVs and parvoviruses, Hayakawa *et al.* analyzed the structure of the replicative intermediate (RI) of BmDNV DNAs by polymerase chain reaction (PCR). The results revealed that, unlike other parvoviruses, BmDNV cannot replicate via self-priming and hairpin-transfer mechanisms. A sequence analysis indicated that VD1-ORF4 encodes a polypeptide that has a conserved group B DNA polymerase II (DNA-pol-B-2) structural domain. The unique feature of these polymerases is that they all use a protein as a primer. The DNA polymerase of BmBDV, a putative protein of 1,105 amino acids with a predicted molecular weight of approximately 127 kDa, contains three 3′-5′ exonuclease domains, five polymerase domains, and an unknown functional domain in its amino-terminal 328 amino acids, has been demonstrated indirectly in a variety of experiments [14–16]. Lacking palindromic sequences at the termini of VD1 and VD2, the ITR could form a panhandle, instead of a hairpin structure. These characteristics are consistent with the view that BmBDV might use a protein-primed replication mechanism [5]. A study of Kojima *et al*. showed that BmDNV encodes a polymerase with DNA-pol-B-2 activity, and that this protein can bind to the CTS of BmDNV. The result suggested that the terminus of the genome encodes a protein that is identical to the terminal protein (TP) of adenoviruses [17].

Adenoviruses and parvoviruses are important animal virus vectors [18, 19], and BmBDV has similar behaviors to these viruses. Because of the restricted number of host species, the recombinant BmBDV virus is limited as a biological insecticide. However, genetic improvement genes could be introduced into the BmBDV genome to create vectors of specific use. For example, silkworm has low digestion efficiency of mulberry leaves due to the lack of cellulase in the midgut. Introduction of cellulase gene into the virus genome and use of it as a vector to transfect the silkworm somatic cells may improve the digestion efficiency. Thus far, infectious virus particles have not been rescued from *Bm*N cells, an ovarian cell line, after transfection with a circular plasmid carrying the BmBDV genome, because the termini of the BmBDV genome were not exposed [20]. However, when full-length VD1- and VD2-containing plasmids were linearized and co-transfected into *Bm*N cells, infectious virus particles could be rescued, although the rescue efficiency was low [21]. BmBDV might use a protein-primed replication mechanism like adenovirus, experiments have proved that some extra adenovirus nts, which affect adenovirus genome replication, are exposed at the termini of the linearized genome [22, 23]. Thus, the low efficiency of BmBDV virus rescue may have been caused by the presence of these extra nts.

In the present study, we adopted methods that have been used to rapidly generate recombinant adenoviruses [24]. First, we cloned the full-length BmBDV genome such that the terminal structure was recoverable by adding a specific endonuclease sequence to the end of the genome. Second, the mCP-encoding gene in the BmBDV genome was fused with the green fluorescence protein (GFP)-encoding gene. Finally, *Bm*N cells were co-transfected with linearized, full-length VD1- and VD2-containing plasmids or linearized, recombinant plasmids expressing mCP-GFP and VP-GFP fusion proteins. PCR data illustrated that there were newly synthesized progeny virus DNA in co-transfected *Bm*N cells. Additionally, western blotting analysis detected the expression of the main structural protein, VP, and green fluorescence was observed in infected cells. Furthermore, reinfected silkworm larvae showed typical densonucleosis symptoms, and more importantly, the direct visualization of green fluorescence allowed us to detect recombinant plasmids that were expressed in the silkworm midgut. Overall, this study provides a platform for researching viral replication mechanisms, viral gene functions, and viral infection mechanisms.

## Methods

### Genome plasmid construction

**Fig. 1:**
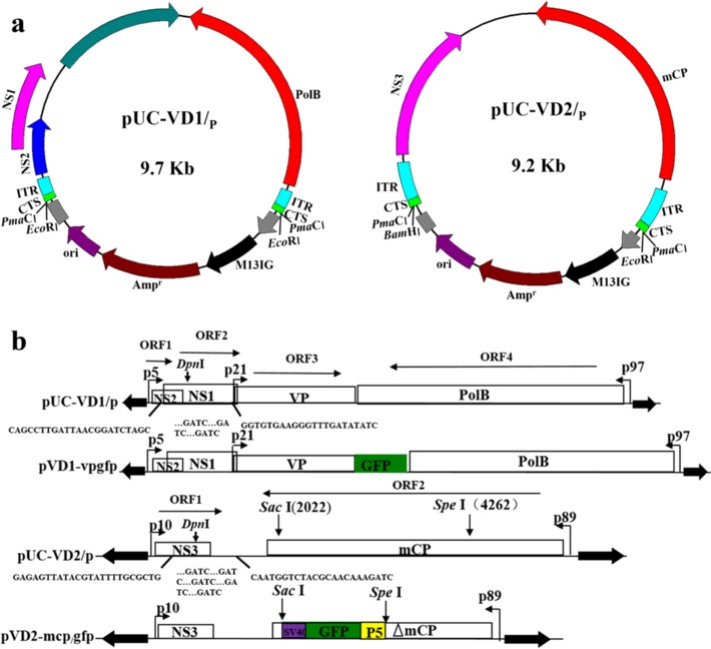
(**a**) Schematic organization of BmBDV genome clone plasmids in which the terminal structure can be reinstated. (**b**). Schematic organization of recombinant BmBDV plasmids pVD1-vp-gfp and pVD2-mcp-gfp

We constructed a genomic clone in which the terminal structure could be reinstated through diagnostic restriction endonuclease digestion. The primers used for constructing the plasmids in this study is listed in Table 2. The restriction endonuclease *Pma*CI recognizes 5′–CACGTG–3′ sequences, and it creates blunt ends with a 5′-GTG–3′ sequence, which is consistent with the CTSs, 5′–GTGTGT–3′, in VD1 and VD2. We constructed two plasmids in our laboratory, pMD18T-VD1 and pUC-VD2, each of which carry portions of the BmBDV genome. We created the specific *Pma*CI recognition sequence by introducing three nucleotides, CAC, at the end of the CTS (5′–GTGTGTGT–3′), and we mutated the two *Pma*CI sites that are present in the ITRs of VD1 (nts 177–182 and 6,361–6,366) from CACGTG to CAGGTG. The functional features of these plasmids are as follows. pMD18T-VD1 contains a *Sac*I site in VD1 (nts 677–680), and we used the *Sac*I site to divide VD1 into two sections, which were subcloned into pUC119 and named pUC-A and pUC-B, respectively. The ClonExpress One-step Cloning Kit (Vazyme Biotech, Nanjing, China) was used to replace the partial terminal sequences (nts 1–182 and 6,361–6,366) by homologous recombination so that nts can be inserted and mutated at these sites. Homologous, recombinant fragments (i.e., 182-bp terminal repeat sequences) were synthesized by PCR using primers with homologous arms. ACAC sequence was introduced into the pUC-A and pUC-B plasmids by the upstream primer, and the *Pma*CI site (CAGGTG) was mutated by the downstream primer. The terminal repeat sequences in pUC-A and pUC-B plasmids were replaced using the ClonExpress One-step Cloning Kit (Vazyme Biotech). The subcloned plasmids were ligated together, which resulted in pUC-VD1/p (Fig. 1a).

**Table 1:** List of abbreviations

Abbreviation	Full name
BmBDV	*Bombyx mori* bidensovirus
ITR	inverted terminal repeats
CTS	common terminal sequence
ORF	open reading frame
mCP	minor capsid protein
ssDNA	Single-stranded DNA
BmDNV-2	*Bombyx mori* Densovirus type 2
pPolB	protein-primed type B DNA polymerase
DNA-pol-B-2	group B DNA polymerase II
pUC-VD1/p, pUC-VD2/p	linearized full-length genomic DNA-containing plasmids
pVD1-*vp*-*gfp*, pVD2-*mcp*-*gf*p	linearized recombinant plasmids

**Table 2:** Primers used for construction of the plasmids in this study

Primer	F/R	Primer sequence (5′-3′)	Ranges of nucleotides (nts)	Instruction
PUC-A	F	GTTGTAAAACGACGGCCAGTGAATTCACGTGTGTGTATACTGGGGCGG	1–182	pUC-VD1
	R	ATCTGTCACTTATCTTGCACCTGACTCTTGATTGTTGCATGTG		
PUC-B	F	ATCTGTCACTTATCTTGCACCTGACTCTTGATTGTTGCATGTG	6361–6366	
	R	GTTGTAAAACGACGGCCAGTGAATTCACGTGTGTGTATACTGGGGCGG		
PUC-C	F	CGAATTCACGTGTGTGTATACTGGGGCGG	1–407	pUC-VD2
	R	CGCTCGAGTACCGCCCCC		
PUC-D	F	CGCTCGAGTACCGCCCCC	5,615–6,022	
	R	CGGGATCCACGTGTGTGTATACTGGGGCGG		
X-VP-pol	F	CGCTCGAGGAGAATATAGAGAAG	2,536–3,533	pVD1-vpgfp
	R	CTCTACTGACCCTGGTACTAAGGAC		
VP-Mu	F	GTCCTACGGGGAGGCTTGTTGGTTTTCAATAAAGTCGACTATTAAAAT	2,536–3,533	
	R	AACAAGCCTCCCGTAGGACTTCCTCCTGGATTAATTCTAG		
EGFP	F	CGGGAGGCTTGTTGGTTTTCAATAAAGTGAGCAAGGGCGAGGAG	–	
	R	GAATAAAACATTTATAAATTTTAATATTACTTGTACAGCTCGTCCATGC		
P5	F	CCGGTACCAATTTATACTTTAAGCC	–	pVD2-mcp_/_gfp
	R	CGCCCTAGGATATAAACTGAGCTTGTATG		
p5-gfp-sv40	F	CGGACTAGTAATTTATACTTTAAGCC	–	
		CGGAGCTCGATGAGTTTGGACAAACCAC

Because there are no *Pma*CI sites in VD2, the CAC sequence was introduced at the end of the genome. A *Sac*I site is present in VD2 (nts 2,015–2,018), and we used the *Sac*I site to divide VD2 into two sections, which were subcloned into pUC119 named pUC-C and pUC-D, respectively. Partial terminal sequences of VD2 (nts 1–407 and 5,615–6,022) were synthesized by PCR so that the CAC sequence could be introduced. An *Xho*I site is present in in the reverse terminal repeat sequence of VD2 (nts 407–410), pUC-C and pUC-D plasmids were digested with *Xho*I. Partial terminal sequences of VD2 were synthesized by PCR using primer design tools. The CAC sequence was introduced into the pUC-C and pUC-D plasmids. Finally, the subcloned plasmids were ligated together, and the resulting plasmid was named pUC-VD2/p (Fig. 1a).

### Recombinant plasmid construction

We constructed a recombinant plasmid in which the *gfp* gene was fused with the *vp* gene. The *gfp* coding sequences were inserted into the 3′ end of ORF3. The *vp* stop codon, TAA, was mutated to GTC, and the initial ATG start codon of the GFP gene was mutated to GAC. The resulting plasmid was named pVD1-*vp*-*gfp* (Fig. 1b). To construct pVD2-*mcp*-*gfp*, a GFP gene expression cassette was inserted into the *Sac*I and *Spe*I sites of the *mcp* gene. The plasmid pT-p5-*gfp*-*sv40*, which was constructed in our laboratory, was used as a source of the GFP gene. The p5 promoter was used to drive NS1 gene expression. Primers were designed for PCR amplification of the p5-*gfp*-*sv40* sequences, and the p5-*gfp*-*sv40* sequences were inserted into the *Sac*I and *Spe*I sites of the *mcp* gene (Fig. 1b).

### Co-transfection of linearized genomes into *Bm*N cells

The plasmids pUC-VD1/p and pUC-VD2/p were extracted and prepared for co-transfections. Supercoiled plasmids (the genomic DNA-containing plasmids pUC-VD1/p and pUC-VD2/p, the recombinant plasmids pVD1-*vp*-*gfp* and pVD2-*mcp*-*gfp*, the negative control plasmid pUC119, and the positive control plasmid pIB-N-GFP, (which is a GFP protein expression vector) that were used for transfection were prepared using the Endo-free Plasmid Purification Kit (Omega Bio-Tek, Norcross, GA, USA). The plasmids were digested with *Pma*CI, purified by ethanol precipitation, and resuspended at a concentration of 1 μg/μl, as previously described [21].

*Bm*N cells were grown at 27 °C in 25-cm^2^ flask cultures containing TC-100 (Life Technologies, Carlsbad, CA, USA) supplemented with 10 % fetal bovine serum (FBS; Gibco, Grand Island, NY, USA) and 1 % antibiotics (penicillin-streptomycin; HyClone Gibco-BRL, Life Technologies, NY, USA). The cell confluency was approximately 80–95 % at the time of transfection. Five groups were set up in the transfection experiments. They included linearized genome-containing plasmids (pUC-VD1/p, pUC-VD2/p), linearized recombinant plasmids (pVD1-*vp*-*gfp*, pVD2-*mcp*-*gfp*), and the control plasmids (pUC119 and pIB-N-GFP). These fragments were prepared by diagnostic restriction endonuclease digestions prior to transfection (usually 3 μg of DNA is needed to transfect one 25-cm^2^ tissue culture flask). Transfections were performed with Cellfectin II Reagent liposomes (Invitrogen, Carlsbad, CA, USA) according to the manufacturer’s instructions (as follows). Three micrograms of *Pma*CI-digested plasmid and 200 μl of serum-free TC-100 medium were mixed. Then, the mixture was incubated for 15 min at room temperature. Then, 10 μl of Cellfectin® II Reagent liposomes reagent and 200 μl of serum-free TC-100 medium were added and incubated for 15 min at room temperature. Finally, the DNA/Cellfectin® II Reagent liposomes reagent mix was incubated for 30 min at room temperature. Meanwhile, *Bm*N cells were washed three times with serum-free TC-100 culture medium. Then, the DNA/ Cellfectin® II Reagent liposomes mixture was added dropwise to the 25-cm^2^ tissue culture flasks, which were placed in a 27 °C incubator. After 5 h, the medium containing DNA/ Cellfectin® II Reagent liposomes reagent mixture was removed, and 10 ml of fresh TC-100 complete medium was added. Four milliliters of fresh TC-100 complete medium was added every 4 d until obvious plaques or cytopathic effects (CPEs) were observed by light microscopy, which usually occurred 2 weeks after the transfection. Green fluorescence emitted by GFP observed by fluorescence microscopy 15 d after the transfections.

### Western blots

Cells were scraped and collected in 50-ml conical tubes and pelleted by centrifugation for 90 min at approximately 3,000 × *g* at 4 °C in a bench-top clinical centrifuge. Then, the supernatant was removed and the cell pellet was collected. Total proteins were extracted from the cells using the Total Protein Extraction Kit (Biyuntian, Shanghai, China). Total proteins were resolved on a 12 % sodium dodecyl sulfate (SDS)-polyacrylamide gel and transferred to a polyvinylidene fluoride membrane. A rabbit anti-VP polyclonal antibody (prepared in our laboratory) and an anti-GFP monoclonal antibody were used as the primary antibodies. Signals were detected using the DAB Kit (Biyuntian). Silkworm midgut fluid, which was infected by BmBDV, served as a positive control. *Bm*N cells that were transfected with pUC119 served as a negative control.

### RNA extraction and reverse transcription (RT)-PCR

Total RNA was extracted from the different groups of infected cells at 48 h. The cells were scraped, collected in 50-ml conical tubes, and pelleted by centrifugation for 90 min at approximately 3,000 rpm at 4 °C in a bench-top clinical centrifuge. The supernatant was removed and the cell pellet was collected. Trizol reagent (Invitrogen) was used to extract the total RNA, which was confirmed by 1 % agarose gel electrophoresis. Any residual DNA was removed by treating it with RNase-free DNase. First-strand cDNA was synthesized using the PrimeScript Reagent Kit with gDNA Eraser (TaKaRa, Shiga, Japan). The NS1 mRNA levels of the five experimental groups were compared. Gene-specific primers were designed using primer design tools Primer5. Primers specific for the NS1 gene were F-NS1 (5’–CGGAATTCATGGAATCGAAGTC–3’) and R-NS1 (5’–CCCTCGAGCCCATAATATTTATTATATAC–3’); the actin gene served as an internal reference.

### Viral DNA detection of transfected cells

Total DNA was extracted from the different groups of infected cells at 48 h after transfection. Cells were scraped, collected in 50-ml conical tubes, and pelleted by centrifugation for 90 min at approximately 3,000 rpm at 4 °C in a bench-top clinical centrifuge. The supernatant was removed and the cell pellet was collected. Total DNA was digested with *Dpn*I to obtain unmethylated DNA, which served as a template in subsequent PCRs. Then, the template was PCR-amplified using the gene-specific primers *Dpn*I-VD1 (5’–CAGCCTTGATTAACGGATCTAGC–3’ for the sense primer and 5’–GATATATCAAACCCTTCACACC–3’ for the antisense primer) and *Dpn*I-VD2 (5’–GAGAGTTATACGTATTTTGCGCTG–3’ for the sense primer and 5’–GATCTTTGTTGCGTAGACCATTG–3’ for the antisense primer). The GATC sequence of plasmid DNA derived from the DH10B strain of *Escherichia coli* was methylated, as opposed to the unmethylated DNA in eukaryotic cells [25], and, thus, could be recognized by *Dpn*I. Five *Dpn*I sites are present in VD1, and three *Dpn*I sites are present in VD2. Gene-specific primers were designed using primer design tools Primer 5.

### Infection of silkworm larvae with viruses isolated from *Bm*N cells

Infected *Bm*N cells were collected from the five experimental groups at 15 d after transfections. Cells were scraped, collected in 50-ml conical tubes, and pelleted by centrifugation for 90 min at approximately 500 × *g* at 4 °C in a bench-top clinical centrifuge. The supernatant was removed and the cell pellet was collected. The cell pellet was washed three times with 1 ml of sterile phosphate-buffered saline (PBS), and the cells were pelleted by centrifugation for 90 min at approximately 500 × *g*. All but 200 μl of the PBS was removed, and the cells were resuspended by vortexing. Two freeze-thaw-vortex cycles were performed to release the viruses from the cells. First and forth instar silkworm (strain QingSong) larvae were fed on mulberry (*Morus alba*) leaves that were smeared evenly with the suspension, and 24 h later, larvae fed on untreated. Every sample contained 30 larvae and the experiment was repeated three times. In addition, larvae fed with untreated *Bm*N cells were used as a negative control.

## Results

### Construction of a recoverable linear genomic plasmid with terminal structures

BmBDV uses a protein-primed replication mechanism. DNA replication requires the exposure of the double-stranded DNA end structure [5, 8, 15]. That is, the circular genomic cone plasmid must revert to the wild-type linear form so that the viral genome can replicate. VD1 and VD2 have 53-nt CTSs that contain a 5’-non-translated region (5’-NTR) with the sequence 5’–GTGTGTGT–3’. *Pma*CI can recognize 5’–CACGTG–3’ sequences, and digestion creates blunt ends that retain the 5’–GTG–3’ sequence. We created the specific recognition sequence of *Pma*CI by introducing three nucleotides, CAC, at the end of the common terminal sequence (5’–GTGTGTGT–3’). To eliminate the two *Pma*CI sites present in the ITRs of VD1 (nts 177–182 and 6,361–6,366), we used site-directed mutagenesis to change their sequence from 5’–CACGTG–3’ to 5’–CAGGTG–3’. We successfully constructed full-length genomic clones of BmBDV, pUC-VD1/p and pUC-VD2/p (Fig. 1a). The terminal structure of BmBDV can be reinstated through diagnostic restriction endonuclease digestion and there is no insertion and deletion.

### Construction of a GFP fusion plasmid

To investigate an exogenous gene expression strategy, we constructed recombinant plasmids in which the *gfp* reporter gene was inserted into the BmBDV genome. The strong viral promoter p21 drives the expression of the major capsid protein-encoding *vp* genes in VD1-ORF3. The *gfp* gene was inserted into ORF3 and fused to the 3’-terminal sequence of the *vp* gene (without its 3’ TAA stop codon). The recombinant plasmid pVD1-*vp*-*gfp*, in which the *gfp* gene was fused with the major capsid protein-encoding *vp* gene, was constructed. The weak viral promoter p89 drives the expression of the *mcp* genes in VD2-ORF2; thus, the abundance of the mCP protein was low in the BmBDV particles. The BmBDV genome contains a small number of genes and it has a compact structure. To avoid disrupting the function of the major viral genes and to improve the expression of the *gfp* gene, we inserted the GFP expression cassette p5-*gfp*-*sv40* into the *mcp* gene. The NS1 gene was transcribed from the p5 promoter, which is a strong early promoter *of* BmBDV [26]. The recombinant plasmid pVD2-*mcp*-*gfp* was constructed, and it contains the p5-*gfp*-*sv40* expression cassette in the *mcp* gene (Fig. 1b).

The plasmids pUC-VD1/p, pUC-VD2/p, pVD1-*vp*-*gfp*, and pVD2-*mcp*-*gfp* were digested by *Pma*CI, and the fragments were the same size as the expected value (data not shown). The full-length BmBDV genomic terminal structure was reinstated with non-redundant nucleotides via restriction endonuclease digestion, and its sequence is consistent with previously reported sequences (GenBank accession numbers DQ017268 and DQ017269).

### *Bm*N cells were infected when co-transfected with a linear genome

**Fig. 2:**
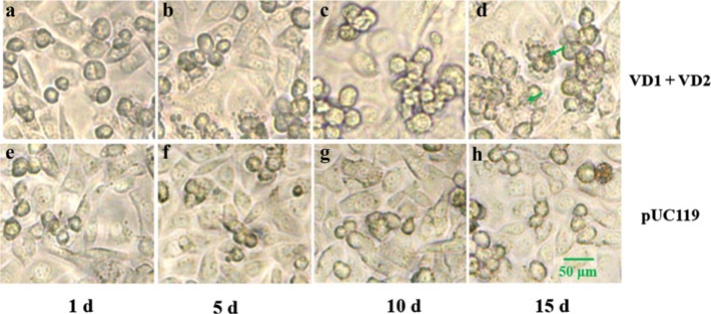
Phase-contrast microscopy images of cells after exhibiting CPEs after co-transfection with full-length genomic fragments. BmN cells were co-transfected with linearized full-length genomic DNA (VD1, VD2) (**a**, **b**, **c**, **d**) or the control plasmid pUC119 (**e**, **f**, **g**, **h**) and observed at the indicated time points. Scale bars represent 50 μm. Arrows indicate CPE structures

To investigate the infectivity of the linear, recombinant BmBDV genome and full-length genomic fragments after transfection, we set up five experimental groups: linearized full-length genomic fragments, linearized recombinant genome (VD1-*vp*-*gfp*) and (VD2-*mcp*-*gfp*), and the control plasmids pUC119 and pIB-N-GFP. Various plasmids were digested by *Pma*CI and transfected into *Bm*N cells. At 24 h after transfection, the experimental group cells (those transfected with VD1 and VD2) were compared with those that were transfected with pUC119. The experimental group cells shape became slightly rounded, and a few detached cells were present. No obvious cytopathic effect (CPE) was observed until 5 days after the co-transfections. On day 10 after the co-transfections, the size of the cell nucleus increased, as did the number of small fragments on the cell surface. On day 15 after the co-transfections, the cells began to break up, their contents were released, and the number of detached cells increased (Fig. 2).

**Fig. 3:**
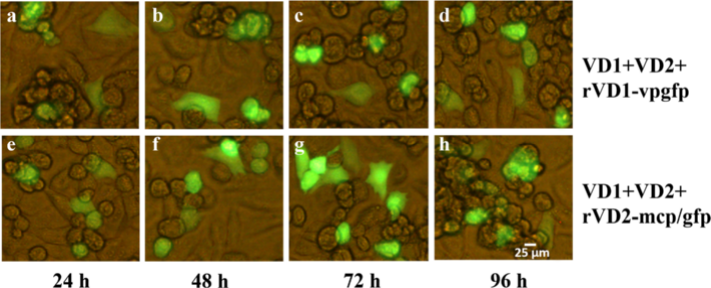
Fluorescence microscopy images of cells displaying CPEs after co-transfection with linearized recombinant plasmids. BmN cells were co-transfected with linearized recombinant genomic DNA (VD1-vp-gfp) (**a**, **b**, **c**, and **d**) or (VD2-mcp-gfp) (**e**, **f**, **g**, and **h**) and observed at the indicated time points. Scale bars represent 25 μm

The linearized recombinant genome (VD1-*vp*-*gfp*) or (VD2-*mcp*-*gfp*) were respectively co-transfected into *Bm*N cells to investigate their infectivity. The direct visualization of green fluorescence allowed us to detect recombinant genome that were expressed in *Bm*N cells. The first fluorescent cells were observed as early as 24 h after transfection by inverted fluorescence microscopy (Fig. 3). However, the transfection efficiency of the experimental group (*Bm*N cells that were transfected with enzyme-digested fragments of the recombinant genome) was low, generally less than 20 %, compared with the control group (*Bm*N cells that were transfected with the pIB-N-GFP plasmid). With increasing culture time, the experimental group cells had a tendency to become round, and more cell surface fragments were observed and some of the cells ruptured (Fig. 3).

### BmBDV genes are transcribed in *Bm*N cells after co-transfection

**Fig. 4:**
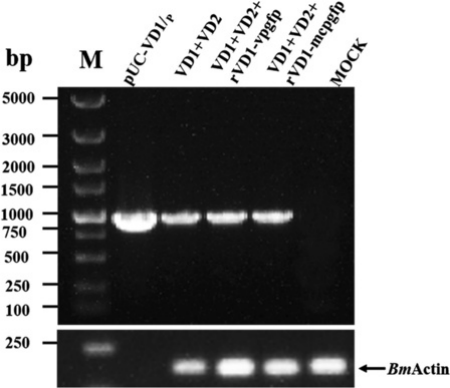
Detection of NS1 mRNA in *Bm*N cells by RT-PCR,the positive control plasmid pUC-VD1/p (lane 1), co-transfection with linearized full-length genomic DNA (lane 2), linearized recombinant genomic DNA(VD1-vpgfp) (lane 3), linearized recombinant genomic DNA(VD2-mcpgfp) (lane 4), or the negative control,non-transformed cells (lane 5). From left to right is lane 1to lane 5

NS1 of BmBDV is a multi-functional, phosphorylated protein that possesses binding ability to ATPase, DNA helicase, and DNAs. NS1 plays an important role in DNA replication, and it shows partial identity to the NS1 protein of parvoviruses [11]. In this regard, NS1 was chosen as the target gene to investigate in viral gene transcription. The *Bm*N cells were transfected with linearized genomic DNA and cultured for 48 h to make sure that the NS1 gene was transcribed. NS1 mRNA was detected by RT-PCR. The 951-bp target band was amplified using the gene-specific primers F-NS1 and R-NS1, and the size of the target band is consistent with the theoretical value (Fig. 4). The actin gene was used as an internal reference, and it yielded a 284-bp product. These results indicate that the NS1 gene was transcribed after BmN cells were transfected with linearized full-length genomic DNA (VD1, VD2), and linearized recombinant genomic DNA (VD1-*vp*-*gfp*) or (VD2-*mcp*-*gfp*).

**Fig. 5:**
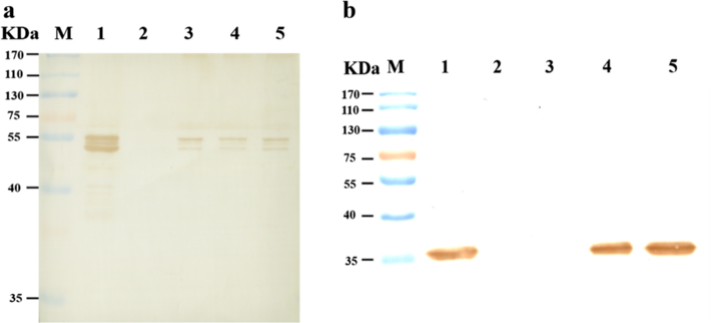
(**a**) Detection of VP expression by western blotting after co-transfection. BmBDV virus particles served as a positive control (lane 1), BmN cells were transfected with plasmid pUC119 as a negative control (lane 2), linearized full-length genomic DNA (VD1, VD2) (lane 3), linearized recombinant genomic DNA(VD1-vp-gfp)(lane 4), and linearized recombinant genomic DNA (VD1-mcp-gfp) (lane 5). (**b**) Detection of GFP expression by western blotting after co-transfection. pIB-N-GFP as a positive control (lane 1), *Bm*N cells were transfected with pUC119 as a negative control (lane 2), linearized full-length genomic DNA ((VD1, VD2) (lane 3), or the linearized recombinant genomic DNA(VD1-vp-gfp) (lane 4) or (VD1-mcp-gfp) (lane 5)

The VP protein is the major structural protein of BmBDV. The expression of VP indicates that BmBDV is likely to be packaged in *Bm*N cells. Linearized genomic DNA was transfected into *Bm*N cells, which were subsequently cultured for 96 h. Total proteins were extracted from co-transfected *Bm*N cells and detected by western blotting using a rabbit anti-VP polyclonal antibody as the primary antibody (Fig. 5a). BmBDV virus particles were used as a positive control group, while *Bm*N cells transfected with pUC119 plasmid served as a negative control group. The total proteins exhibited two bands of approximately 55 and 51 kDa in the positive control and experimental groups (Fig. 5a), but these bands were not detected in the negative control group. Western blot data also indicated that the BmBDV structural protein VP was expressed in *Bm*N cells after co-transfection. GFP was used as a marker for exogenous genes expression. To confirm that GFP was expressed in *Bm*N cells, total protein was extracted from *Bm*N cells and analyzed by western blotting using an anti-GFP monoclonal antibody as the primary antibody (Fig. 5b). *Bm*N cells transfected with pIB-N-GFP served as a positive control group, and cells transfected with pUC119 served as a negative control group. In the positive control and recombinant plasmid experimental groups, the total protein yielded a 35-kDa band, which was not detected in the negative control and full-length genomic plasmid experimental groups (Fig. 5b). GFP was also expressed in *Bm*N cells after co-transfection.

### Detection of progeny virus DNA by PCR after co-transfection

**Fig. 6:**
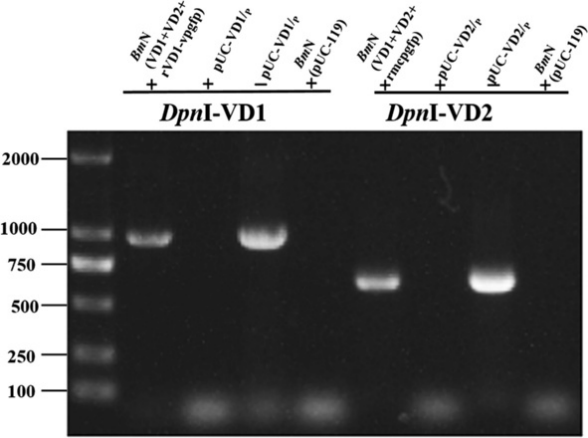
Detection of progeny virus DNA by PCR. *Dpn*I-VD1 and *Dpn*I-VD2 were used as gene-specific primers for PCR amplifications. BmN (VD1 + VD2) is the total DNA of BmN cells that was co-transfected with linearized full-length genomic DNA- (VD1, VD2); BmN (vp-gfp) is the total DNA of BmN cells that were co-transfected with the linearized recombinant genomic DNA (VD1-vp-gfp); BmN (mcp-gfp) is the total DNA of BmN cells that were co-transfected with the linearized recombinant genomic DNA (VD2-mcp-gfp); pUC-VD1/p and pUC-VD/p served as templates for the positive control group; BmN cells transfected with pUC119 served as the negative control group. The " + " and " − " denote DpnI-treated and untreated templates

To determine whether the BmBDV genome could be synthesized in *Bm*N cells after transfection (linearized, full-length genomic DNA-containing plasmids and linearized recombinant plasmids), total DNA was extracted and digested with *Dpn*I [27, 28]. Two pairs of gene-specific primers, *Dpn*I-VD1 and *Dpn*I-VD2, were used for PCR amplifications. *Dpn*I recognizes the 5’–GATC–3’ sequence. The template DNA that was extracted from *Bm*N cells and digested with *Dpn*I yielded 981-bp and 633-bp fragments by PCR, which are consistent with the expected band sizes (Fig. 6). Using full-length genomic DNA-containing plasmids

(pUC-VD1/p and pUC-VD2/p), which were not digested with *Dpn*I, as templates resulted in the amplification of the same size bands. When *Dpn*I-digested pUC-VD1/p and pUC-VD2/p plasmids and the negative control group (*Bm*N cells transfected with pUC119) were used as templates, the same size bands could not be amplified (Fig. 6). The result indicated that progeny virus DNA was present in *Bm*N cells after co-transfection. Thus, the DNA should be that of the progeny viruses that were synthesized in *Bm*N cells.

### Determination of whether BmBDV virions are produced by silkworm larvae that were infected with viruses from *Bm*N cells

**Fig. 7:**
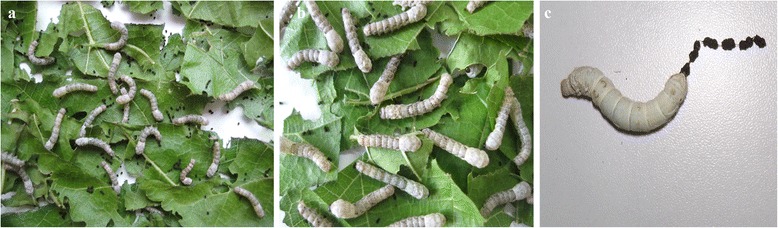
The symptoms caused by reinfecting silkworm larvae that were fed a virus- infected cell suspension (**a**); the negative control experiment in which silkworms were fed a virus-free cell suspension (**b**); silkworms in the experimental group produced clusters of bead-like feces (**c**)

To determine whether the BmBDV virions are produced successfully in *Bm*N cells, we set up two reinfection groups. First instar silkworm (strain QingSong) larvae were fed on a virus-infected cell suspension and served as the experimental group, while silkworms that were fed on a virus-free cell suspension served as the control group. By the second instar, the silkworm larvae of the experimental group started showing symptoms, such as moving slowly and exhibiting a decreased food intake. The experimental group was compared to the control group, and the ecdysis time of larvae in the experimental group occurred much later than in the control group (Fig. 7). Additionally, the experimental group exhibited slow growth and produced clusters of bead-like feces (Fig. 7). Then, a few days later, the thoracic regions of experimental group became translucent due to tissue degradation. These results indicate that viral genome was rescued.

### Determination of whether recombinant BmBDV virions could be produced by silkworm larvae that were infected with viruses from *Bm*N cells

**Fig. 8:**
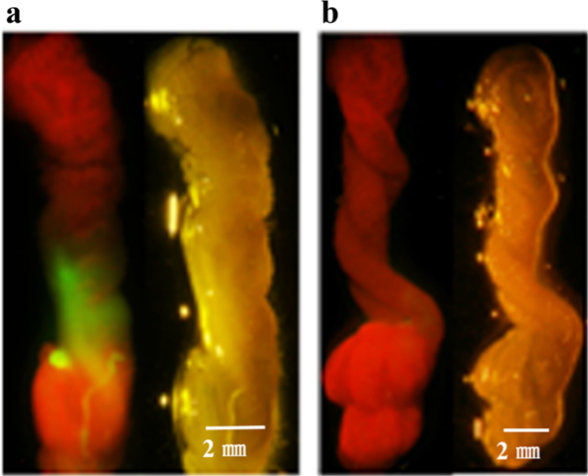
Expression of green fluorescence in the midgut of silkworm larvae. (**a**) Silkworm larvae were infected with recombinant viruses that were isolated from infected cells that were co-transfected with the linearized recombinant genomic DNA(VD1-vp-gfp); (**b**) silkworm larvae were infected with cell supernatants (obtained from cells that were co-transfected with pIB-N-GFP) as a negative control. The left side of each panel is under fluorescence and the right side is using bright field. Scale bars represent 2 mm

GFP is a robust marker for identifying recombinant vector-transfected cells *in vitro* and *in vivo*. To determine whether recombinant BmBDV virions could be produced successfully in *Bm*N cells, we fed first instar silkworm (the QingSong strain) larvae on mulberry leaves treated with cell supernatants that were collected from infected cells (cells that were co-transfected with linearized recombinant genomic DNA (VD1-*vp*-*gfp*) or(VD2-*mcp*-*gfp)*. Typical symptoms caused by reinfection could be observed, and midgut tissues that were invaded by BmBDV were yellow and thin, and green fluorescence could be observed (Fig. 8). These results indicate that the BmBDV genome was packed into viral particles and formed recombinant BmBDV virions following transfection. The recombinant viruses show similar behaviors to the native BmBDV.

## Discussion

BmBDV invades the columnar cells of the larval midgut epithelium and causes chronic densonucleosis disease in silkworms, which results in great economic losses to the sericulture industry. In the present study, *Bm*N cells were co-transfected with the circular plasmids pMD18T-VD1 and pUC-VD2, and neither BmBDV genome replication nor protein expression in *Bm*N cells could be detected (data not shown). As described previously, BmBDV might use a protein-primed replicating mechanism that is similar to that of adenoviruses [5, 8, 15]; thus, it is necessary to expose the terminal region of the linear genome for the protein-priming DNA replication mechanism to work. Therefore, we constructed a BmBDV full-length genomic clone, and the linear genomic terminal region was exposed by digestion, so that a large number of double-stranded, linearized viral genomes could be obtained. The VP protein is the major structural protein of BmBDV, and GFP is a robust marker for the expression of exogenous genes. BmBDV genomic fragments were co-transfected into *Bm*N cells, and the expression of VP and GFP was detected by western blotting. Three different proteins (with sizes of approximately 55, 53, and 51 kDa, which were consistent with the theoretical values of VP1, VP2, and VP3, respectively) were detected by western blotting using an anti-VP polyclonal antibody. VD1-ORF3 encodes VP1, VP2, and VP3, which are expressed via a leaky scanning mechanism. Additionally, a 35-kDa protein, which is consistent with the predicted size of GFP, was identified by western blotting using an anti-GFP monoclonal antibody. Western blot data also indicated that the VP protein and GFP were expressed in *Bm*N cells. The expression of the VP protein provides a foundation for packaging virus particles, while the expression of GFP raises the possibility of using BmBDV as a virus vector. mRNA and DNA were extracted from *Bm*N cells after co-transfection, and using gene-specific primers and mRNA and DNA as template, we PCR-amplified the target bands, the sizes of which were consistent with their theoretical values. PCR data also indicated that the progeny BmBDV viral DNA was replicated, and that mRNA was transcribed in *Bm*N cells after co-transfection. These results indicate that the progeny virus DNA was synthesized in *Bm*N cells.

To determine whether infectious BmBDV virions could be produced in *Bm*N cells, we fed first instar silkworm larvae on supernatants of infected cells that were co-transfected with linearized full-length genomic DNA (VD1, VD2). By the second instar, silkworms started showing typical symptoms of densonucleosis. The result illustrated that co-transfected *Bm*N cells produce infectious virus particles that can infect silkworm larvae, thereby demonstrating that the viral genome was rescued. To determine whether recombinant BmBDV virions could be produced in *Bm*N cells, we fed first instar larvae on infected cells that were co-transfected with the linearized recombinant genomic DNA (VD1-*vp*-*gfp* or VD2-*mcp*-*gfp)*. By the fourth instar, silkworms started showing typical symptoms of densonucleosis, and green fluorescence was observed in their midgut tissues, which were yellow and thin. These results clearly show that infectious recombinant virions could be rescued from *Bm*N cells after co-transfection. Furthermore, the results also provided evidence that the replication mechanism of BmBDV is similar to that of adenoviruses, and that a linear genome and an exposed genome terminus was needed.

BmBDV is a chronic virus, and the disease duration is 7–12 d; sick silkworms die after 10–20 d [29]. *Bm*N cells are not its natural hosts [2], and the virus grows slowly, and their transfection efficiency is low, generally less than 20 % compared with that of Sf9; thus, virus assembly and replication are very limited in *Bm*N cells. Sf9 cells are a high transfection efficiency line, and using the same method, when Sf9 cells were co-transfected with linear, full-length genomic fragments, neither protein nor progeny DNA synthesis could be detected by western blotting or PCR, respectively (data not shown). Thus, BmBDV can specifically infect silkworm larvae midgut columnar cells, and it also has relatively strong specificity for *Bm*N cell lines.

## Conclusions

In summation, linear viral genome fragments were transfected into cells by the linear co-transfection method, which breaks the limit of the BmBDV-specific recognition of midgut tissue cells, and we constructed an experimental detection model for recombinant linear BmBDV genome transfection. Furthermore, our study provides a model to further investigate the relationship between BmBDV and silkworms, and lays the foundation for studying viral replication mechanisms, viral protein expression, and BmBDV regulatory mechanisms.
